# Expanding the Legacy: The National Library of Medicine Michael E. DeBakey Lecture in the History of Medicine

**DOI:** 10.14797/mdcvj.1154

**Published:** 2022-12-06

**Authors:** Jeffrey S. Reznick, Kenneth M. Koyle

**Affiliations:** 1 National Library of Medicine, National Institutes of Health, Bethesda, Maryland, US

**Keywords:** DeBakey, fellowship, research, history of medicine

## Abstract

In our 2021 article published in this journal, we described the development, historical significance, and impact of the National Library of Medicine (NLM) Michael E. DeBakey fellowship in the History of Medicine. This article focuses on a key part of the fellowship, the NLM Michael E. DeBakey Lecture in the History of Medicine, by explaining how this annual program advances historical scholarship and promotes awareness of DeBakey’s legacy and his support of the world’s largest biomedical library, whose collections are appreciated by researchers worldwide. The annual DeBakey Lecture provides a platform for a selected DeBakey fellow to share and expand on their fellowship research, connecting that research and the fellow’s story with a global audience through a videocast, a permanently and freely available archived lecture, a research-based blog post, and an associated blog interview. The lectures have covered topics about DeBakey himself, his influence on the world, and new research that reflects his historical interests. The library’s support of this impactful program, like the Michael E. DeBakey fellowship overall, testifies to its commitment to expanding the legacy of DeBakey hand in hand with its commitment to serving scientists and society in the 21st century.

## Introduction

The National Library of Medicine (NLM) presents an annual Michael E. DeBakey Lecture in the History of Medicine, a key part of the NLM Michael E. DeBakey fellowship in the History of Medicine, which further expands public awareness of DeBakey and his appreciation of well-rounded careers built on both scientific and humanistic understanding.^1^ Delivered by a selected NLM DeBakey fellow, the DeBakey Lecture itself is a cornerstone of the annual NLM History Talks program, which promotes awareness and use of NLM and other historical collections for research, education, and public service in biomedicine, social sciences, and humanities. All NLM History Talks are livestreamed globally, closed-captioned, permanently archived, and freely available via National Institutes of Health (NIH) Videocasting.^2^

Since the first DeBakey Lecture in 2017, the annual lectures have covered topics about DeBakey himself, his influence on the world, and new research that reflects his humanistic view of medicine. Together, these lectures have garnered nearly 2,000 views through their archived videocasts. Associated interviews with the speakers, published on *Circulating Now*, the blog of the NLM History of Medicine Division, have been read more than 8,000 times.^3^ Additionally, the posts authored by NLM DeBakey fellows themselves for *Circulating Now*, based on their completed research, have been read more than 31,000 times. Taken together, this data as well as the high scholarly quality of every lecture offers a measure indicating how these initiatives overall have raised public awareness of the name and legacy of DeBakey.

**Figure 1:**
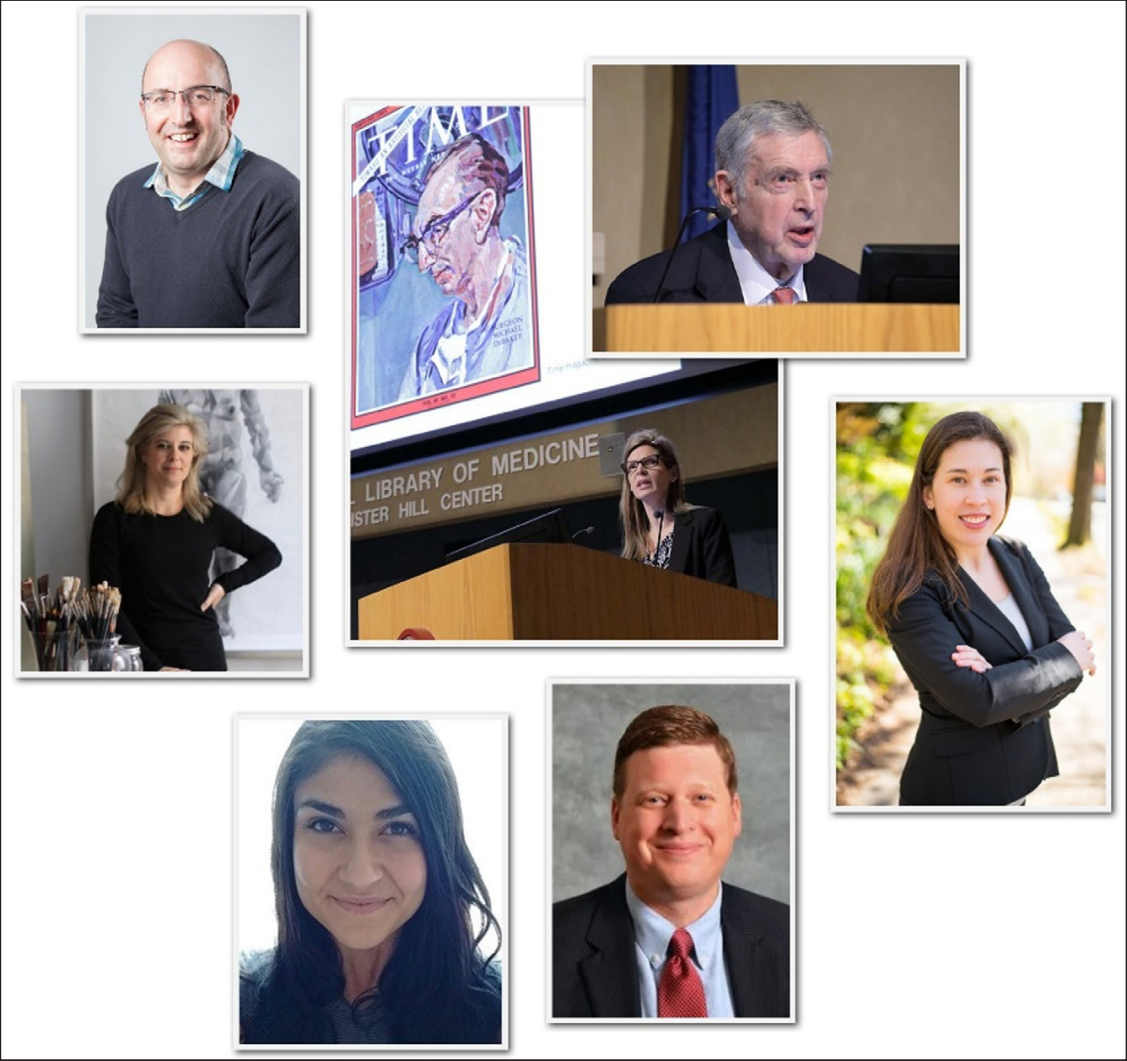
National Library of Medicine DeBakey Lecturers 2017-2022 (clockwise from upper left): Matthew Stibbe, Shelley McKellar, George P. Noon, Heidi Morefield, Andrew T. Simpson, Sara Farhan, and Allison Hill-Edgar. Courtesy National Library of Medicine, National Institutes of Health.

Since its inception, the annual Michael E. DeBakey Lecture in the History of Medicine has provided opportunities for selected DeBakey fellows to give a presentation about their research in the collections of the NLM. The first few lectures understandably focused on DeBakey’s life and medical career. After all, DeBakey himself was so dynamic and multifaceted that his life provided ample material for fascinating research and exploration. As great as his legacy of personal accomplishment may be, perhaps an even greater legacy was his support of scholarly pursuits beyond his own work. Prolific as a teacher, DeBakey was also a lifelong student of the world, observing, exploring, and learning through his travels and his thoughtful engagement with people he encountered along the way. As the lecture series expands to cover subjects DeBakey himself may have never contemplated, there can be little doubt that he would have been as interested and intrigued by these subjects as are the researchers presenting the lectures (Figure 1).

## 2017: Intentional Impact

The inaugural NLM DeBakey Lecture, held on March 21, 2017, involved two preeminent speakers: Western University’s Shelley McKellar, PhD, the Jason A. Hannah Chair in the History of Medicine and associate professor with joint appointment in the Department of Surgery, and Baylor College of Medicine’s George P. Noon, MD, Professor of Surgery in the Michael E. DeBakey Department of Surgery, who had studied under DeBakey and later worked with him as a member of his surgical team at Baylor.^4^

In her lecture, “‘Intentional Impact:’ The Legacy of Michael E. DeBakey Beyond the Operating Room,” McKellar explained that DeBakey was more than a pioneering surgeon; he was a seminal figure who inspired a whole generation, bringing people together and innovating in new and interesting ways. McKellar was “intrigued by what he did in the operating room and how he translates expertise in the OR into political and policy capital.”^4^ Translating personal expertise into beneficial public action is an art, and DeBakey was a master. He worked with intentional, deliberate focus, “directing his time and energy to things that need improvement,” pushing policies and widescale changes that would result in overall better patient care.^4^ DeBakey was featured in *Time* magazine three times, was often headlined in newspapers and other media around the world, and was undoubtedly the most famous medical practitioner of his generation. But this fame was not merely based on his expertise as a surgeon, nor was it something he sought for his own ego or personal gain. He was, in McKellar’s words, “intentionally impactful when it came to things like medical research, education, patient care, and more.”^4^ He earned his reputation through hard work and unparalleled skill, and he used his standing as a world-renowned medical authority to improve healthcare writ large.

Noon, in his complementary lecture, “A Brief Look at Michael E. DeBakey’s Role in Establishing the National Library of Medicine as It Is Today,” shared stories about DeBakey’s seven decades of support for the NLM, stemming from his research in the library as an Army surgical consultant during World War II and continuing into the 21st century. “Dr. DeBakey felt the NLM was a national and international treasure that should be nurtured and supported as needed to maintain its excellence,” Noon explained.^5^ In fact, DeBakey was so concerned about the future of the library that he joined the Association of Honorary Consultants to the Army Medical Library (NLM’s predecessor institution) in 1946 and advocated successfully for transforming the Army Medical Library into a national medical library serving the broader public beyond the armed services. Later, he personally lobbied senators Lister Hill and John F. Kennedy to craft legislation that officially established the National Library of Medicine in the 1950s. DeBakey remained involved through his membership on the Board of Regents and participation in other NLM programs throughout his life.^5^

The NIH community warmly received the inaugural NLM Michael E. DeBakey Lecture in the History of Medicine. In its article, “NLM Commemorates Heart Surgeon DeBakey,” the *NIH Record* noted that although DeBakey had been a household name across America in the 1960s, “There’s a younger generation that likely has never heard of him.”^6^ The article concluded that the legacy of this “world-renowned surgeon, innovator, educator and medical research advocate” would therefore be carried forward, and even expanded, through the annual NLM DeBakey Lecture.

## 2018: Transplanting Technology

The second annual NLM Michael E. DeBakey Lecture in the History of Medicine, held May 24, 2018, was delivered by Heidi Morefield, a 2017 NLM Michael E. DeBakey Fellow in the History of Medicine and a doctoral candidate in the Department of the History of Medicine of The Johns Hopkins University.^7^ Morefield spoke on “Transplanting Technology: Dr. Michael DeBakey and Cold War Technology Transfer.” Following extensive research in DeBakey’s diaries, letters, and personal papers at NLM, Morefield discussed DeBakey’s worldwide travels as a medical ambassador and a “steward of medical technology” during the Cold War.^8^ His status and reputation as the world’s preeminent surgeon gave him unfettered access to places that would have been restricted to other Westerners, particularly the Soviet Union and China. DeBakey’s detailed notes about his visits to foreign countries and his exposure to their medical systems showed a sincere appreciation for different, often low-tech approaches to providing health care. He demonstrated Western methods, including his own inventions, and watched attentively as his hosts shared their techniques. “For him,” Morefield explained, “technology was simultaneously a medium and a message, a site of teaching and learning.” In Morefield’s assessment, “[DeBakey] saw medicine as a form of humanitarianism which had the potential to bring peace to the world by improving individual lives.”^8^

## 2019: Influence in the Business of Health Care

The third annual NLM Michael E. DeBakey Lecture, held May 23, 2019, was delivered by Andrew T. Simpson, PhD, a 2017 NLM Michael E. DeBakey Fellow in the History of Medicine and assistant professor in the Department of History of Duquesne University.^9^ Simpson spoke on “Dr. Michael E. DeBakey and His Influence in the Changing Business of Healthcare and the Delivery of American Medicine.” In his lecture, Simpson examined DeBakey’s role as a “medical entrepreneur” and a key figure in the development of medicine as an enterprise that factors in both public good and commercial viability.^9^ DeBakey cultivated a well-deserved reputation as an advocate for public access to health care, but he also understood that for the healthcare industry to flourish, it required a careful balance between benevolent access and medical capitalism, between the “medical free market and how care is paid for.”

In examining DeBakey’s career through this entrepreneurial lens, Simpson identified three main facets: expansion of specialty medical care abroad and at home, developing and marketing medical devices and innovations, and DeBakey’s vision of what a national healthcare system should look like.^9^ He addressed these facets in his lecture by merging the perspectives of history, economics, and public policy. In his analysis, Simpson found that DeBakey was “a medical administrator and a physician who had a far more complex understanding of where the American healthcare system and its commercial imperative was heading during these decades than is often discussed.”^9^

## 2020: Debakey in Baghdad and Beirut

The fourth annual NLM Michael E. DeBakey Lecture, held September 9, 2020, was delivered by Sara Farhan, PhD, a 2019 NLM Michael E. DeBakey Fellow in the History of Medicine and assistant professor of history at the American University of Sharjah.^10^ Farhan spoke on “DeBakey in Baghdad and Beirut: The Internationalization of Surgical Education, 1945-1970.” Exploring yet another aspect of Michael DeBakey’s multifaceted career, Farhan described his role as a medical educator and de facto ambassador in the Middle East. She argues that “the development of medical education operated along intricate and complex symbiosis where the sharing of knowledge and research transcended national borders.”^11^ Being of Lebanese descent, DeBakey had a familial connection with the Middle East and was viewed in Arab countries as a part of their medical legacy and a source of dignity and pride. In 1954, he attended the 4th Annual Middle East Medical Assembly in Beirut, following the conference with a month-long tour of medical schools in the region, teaching, lecturing, and examining the medical education programs. As Farhan noted during her lecture, DeBakey’s “footprints were in the Middle East prior to his visit.”^10^ He found that the medical colleges in Syria, Lebanon, and Iraq were studying and incorporating his own findings along with those of other prominent medical professionals. “Surgeons in the Middle East were not working in isolation,” Farhan surmised. “On the contrary, they were part of an intricate network of professionals who celebrated the exchange of knowledge and embraced new development in the field.”^10^ DeBakey’s Lebanese heritage and his willingness to travel and engage with medical schools in the region helped to facilitate an exchange of medical knowledge and education between the Middle East and the United States that continues to this day.

Shortly after Farhan’s lecture, the NIH community again expressed its appreciation of the annual NLM DeBakey Lecture. In its article “Fellow Explores DeBakey’s Far Reaching Legacy,” the *NIH Record* acknowledged that beyond sponsoring the DeBakey fellowship in the History of Medicine and housing “an archive of DeBakey’s seminal papers,” the NLM “further honors his legacy” by hosting the annual DeBakey Lecture. Among Farhan’s most important conclusions, the *NIH Record* suggested, was the fact that “DeBakey celebrated the knowledge exchange shared between the Middle East and the United States. He did not see himself as superior to medical professionals in the region, but as their equal.” To this Farhan added that “his relationships with medical professionals across the Middle East continued to flourish for decades until his death in 2008.”^12^

## 2021: Dissecting Gender

The fifth annual NLM Michael E. DeBakey Lecture, held June 3, 2021, was delivered by the artist and independent scholar Allison Hill-Edgar, MD, MFA, 2020 NLM Michael E. DeBakey Fellow in the History of Medicine and a lecturer at the Fenimore Art Museum.^13^ Hill-Edgar spoke on “Dissecting Gender: Reframing Anatomical History Through the Female Body.” Noting that in anatomical texts throughout history, the “depictions of women, when included, were presented according to certain recurring themes and stereotypes that remain present in much of mainstream and medical culture today,” Hill-Edgar analyzed anatomical drawings and images in medical books through the centuries to expose the cultural dynamics that contributed to the view of the female body as the reproductive “other” to the universal male figure.^14^

Having used the NLM collections to examine early Islamic anatomical illustrations, multiple editions of the famous *Gray’s Anatomy* from its original publication to the present version, an 18th-century obstetrical atlas where she found surprising images, and other historical texts, Hill-Edgar cited these examples to frame several themes in the history of female representation in medical texts and relate those themes to contemporary issues in medicine and society.

Hill-Edgar offered the first DeBakey Lecture that was not focused on the work and life of DeBakey himself, demonstrating the capacity of the lecture series to expand DeBakey’s legacy by addressing important new areas of historical research that are possible due to his advocacy for establishing a national medical library with extensive historical collections open to all who wish to consult them.

## 2022: A Laboratory of Humanitarianism

The sixth annual NLM Michael E. DeBakey Lecture, held May 5, 2022, was delivered by Matthew Stibbe, PhD, 2019 NLM Michael E. DeBakey Fellow in the History of Medicine and professor of Modern European History at Sheffield Hallam University.^15^ He spoke on “A Laboratory of Humanitarianism: Military and Civilian Captivity during the First World War.” Drawing on his research in the historical collections of the NLM, including the papers of Michael E. DeBakey, Stibbe explained that the subject of global wartime captivity has overlapping military and civilian dimensions and how studying it reveals important perspectives on humanitarian activism, particularly how it “became more reflexive as the war continued.” Stibbe explained that some types of assistance could “actually make things worse” for individuals held in captivity, while other forms could “have unintended side effects, prolonging a situation when the only humane alternative [was] to end it as soon as possible.”

Notably, in presenting his research, Stibbe made clear his appreciation of DeBakey’s support of the library, and particularly DeBakey’s assertion that the holdings of the institution pertained not only to the military and war but also to civilian life, indeed humanity in all of its complexity. Stibbe’s research on the history of wartime captivity and the various approaches to humanitarian assistance, therefore, served to expand DeBakey’s legacy of preserving information from the past to help inform decisions in the present—a legacy that made his research project possible, along with many more, today and tomorrow.^16^

## Conclusion: The Future of the Legacy

On April 28, 2008, President George W. Bush joined members of Congress in awarding Michael DeBakey the Congressional Gold Medal. “It was Hippocrates,” Bush observed, “the author of the doctor’s sacred oath, who said, ‘Wherever the art of medicine is loved, there also is love of humanity.’ Truer words could not be spoken of Michael DeBakey.”^17^ These words ring true today as the National Library of Medicine expands DeBakey’s legacy of scientific inquiry built on a foundation of humanist morals. DeBakey demonstrated through his life and his work that medicine is more than a purely scientific endeavor; it is the aggregation of scientific knowledge, ethics, morals, and human solicitude. The scholars who present the annual lecture in his name address all of these aspects, and in so doing assure that his legacy will be carried forward for generations to come.
